# Gradual extinction reduces reinstatement

**DOI:** 10.3389/fnbeh.2015.00254

**Published:** 2015-09-15

**Authors:** Youssef Shiban, Jasmin Wittmann, Mara Weißinger, Andreas Mühlberger

**Affiliations:** Department of Clinical Psychology and Psychotherapy, Institute of Psychology, University of RegensburgRegensburg, Germany

**Keywords:** gradual extinction, virtual reality, pavlovian fear conditioning, skin conductance response, startle response, contingency ratings

## Abstract

The current study investigated whether gradually reducing the frequency of aversive stimuli during extinction can prevent the return of fear. Thirty-one participants of a three-stage procedure (acquisition, extinction and a reinstatement test on day 2) were randomly assigned to a standard extinction (SE) and gradual extinction (GE) procedure. The two groups differed only in the extinction procedure. While the SE group ran through a regular extinction process without any negative events, the frequency of the aversive stimuli during the extinction phase was gradually reduced for the GE group. The unconditioned stimulus (US) was an air blast (5 bar, 10 ms). A spider and a scorpion were used as conditioned stimuli (CS). The outcome variables were contingency ratings and physiological measures (skin conductance response, SCR and startle response). There were no differences found between the two groups for the acquisition and extinction phases concerning contingency ratings, SCR, or startle response. GE compared to SE significantly reduced the return of fear in the reinstatement test for the startle response but not for SCR or contingency ratings. This study was successful in translating the findings in rodent to humans. The results suggest that the GE process is suitable for increasing the efficacy of fear extinction.

## Introduction

Anxiety disorders are the most common cases of mental disorders (Merikangas et al., 2010) and can be treated with exposure therapy, which has proven to be an effective strategy for treating fear (Hofmann and Smits, 2008). Exposure therapy is presumably based on extinction (Pavlov, 1927): repeated presentation of a previously learned threat stimulus without negative consequences. In most cases, this approach leads to a temporary reduction of anxiety (Vervliet et al., 2013). The return of such extinguished anxiety is a widespread problem. For clinicians, this frequent relapse after a successful extinction is a big challenge, which is why it is so crucial to understand the mechanisms of extinction.

There are different approaches to prevent a relapse: massive extinction treatment (Denniston et al., 2003), multiple context exposure (Shiban et al., 2013), renewal testing in the presence of a retrieval cue from extinction (Brooks and Bouton, 1993) and gradual extinction (GE; Gershman et al., 2013). The last method, GE, includes a modified extinction process, during which the aversive stimulus (US) is not completely absent, but the frequency of its occurrence is gradually reduced. The recent study with rats by Gershman et al. (2013) provides promising results in support of the efficacy of this method. In the following study, we applied these findings to a human sample. To understand the basic assumptions of this approach, a more detailed look into extinction processes is required. Extinction learning is believed to extinguish the conditioned response by presenting the conditioned stimuli (CS) without the unconditioned stimulus (US) during a number of trials.

A traditional model of Pavlovian conditioning (Rescorla and Wagner, 1972) asserts that learning is the modification of associations between the CS and US. Therefore, fear conditioning is reinforcement and extinction is weakening of the initial association. More modern approaches have found evidence that the initial fear memory does not tend to weaken: a whole new memory inhibiting the initial CS-US association is formed (Bouton, 2004). Inhibitory learning is characterized by retained original fear memory which competes with the new model. Bouton (2004) considers the fact that animals learn context, not just CS-US associations. Contextual and temporal clues during the learning process are crucial for saving new information. It would be a mistake to assume that anxiety is just a result of the association strength between the CS and US. In fact, two memories seem to coexist after extinction: an excitatory CS-US association and an inhibitory CS- no US association. The excitatory association causes a fear reaction, while the inhibitory association prevents the reaction entirely.

An approach for enhancing the inhibitory associations, consequently making extinction learning more effective, was made by Craske et al. (2014). According to them, exposure optimization strategies include: (1) expectancy violation; (2) deepened extinction; (3) occasional reinforced extinction; (4) removal of safety signals; (5) variability; (6) retrieval cues; (7) multiple contexts; and (8) affect labeling. The first strategy, expectancy violation, is based on the assumption that a mismatch between expectancy and experience is crucial for learning (Rescorla and Wagner, 1972). Expectancy violation—the discrepancy between an anticipated outcome and a real outcome concerning the frequency or intensity of aversive stimuli during the extinction phase—should be maximized, so that the inhibitory association can be strengthened.

A similar concept, which acknowledges the creation of a new “extinction” memory as the reason for the return of fear, is the “state” concept. The extinction process is postulated to be perceived as a new state of the world (Redish et al., 2007), which results in forming a new memory. Consequently, two competing memories co-exist depending on the learning context: the conditioning state and the extinction state.

Why does this new state emerge? The absence of the aversive stimulus during a traditional extinction phase signals a change to take place; expectations are violated and learning occurs (Rescorla and Wagner, 1972). According to Redish et al. (2007), prediction errors might be misinterpreted as indicators for a new state. Therefore, a new “no-fear” memory—which includes the new associations—is formed and starts competing with the retained original fear memory.

If massive prediction errors function as instructive signals for a new state, a fear memory could be modified by prediction errors that are small enough not to induce the formation of a new memory, but still massive enough to drive learning (Gershman et al., 2013). So, as a hypothesis, this would lead to a more efficient extinction of fear and prevention of relapses when compared to the standard extinction (SE) process.

A recent study with rats by Gershman et al. (2013) provides strong evidence in support of this hypothesis. The investigators demonstrated in two Pavlovian fear conditioning experiments that gradually reducing the frequency of the aversive stimuli, rather than eliminating them abruptly, prevents the return of fear.

The aim of the present experiment was to apply these findings from rats to humans. Based on the classical Pavlovian conditioning paradigm, fear is learned in an acquisition phase, and is afterwards extinguished in an extinction phase, which differed for the two experimental groups. The Standard group took part in the original extinction process, whereas the Gradual group underwent GE, for which the occurrence of the US was gradually eliminated so that the prediction error was high enough to drive learning, but not high enough to cause the creation of a new memory. Thus, weakening the original fear memory should be ensured. The efficiency of GE for extinguishing fear and preventing the return of fear was measured by reinstatement on a subjective level (contingency ratings) as well as on a physiological level (startle response and skin conductance response, SCR). Moreover, the experiment was conducted in a virtual reality (VR), which has been proven an efficient tool to investigate basic processes of conditioning (Glotzbach et al., 2012) and therapy research (Shiban et al., 2013).

## Materials and Methods

### Participants

Thirty-one volunteers were recruited through advertisements at the University of Regensburg. Recruitment took place from April to September 2014. After the participants gave their written consent, the exclusion criteria (spider phobia, age <18 and >50, current involvement in psycho- or pharmacotherapy, neurologically related diseases, a history of psychotropic drug use, color blindness and hearing disorders) were assessed with a demographic questionnaire. All 31 participants (80.6% female, age ranged between 18 and 41, *M* = 24.0, *SD* = 4.69) were students at the University of Regensburg and obtained credit points as reimbursement for their participation. Participants were pseudo-randomly divided (depending on survey date) into two groups based on the respective extinction process (described in detail in the “Procedure” Section). The two groups did not differ significantly in the number of participants, age, gender or in their FSQ and STAI scores (see Table 1). The Ethics Committee of the University of Regensburg approved the study.

**Table 1:** **Demographic variables and questionnaire data**.

	Standard group			Gradual group		
	*n*	*M*	*SD*	*n*	*M*	*SD*	*df*	*t*	*p*
Age	15	24.5	3.79	16	23.6	5.59	29	0.530	0.600
**Questionnaires**
STAI-State1 (20–80)	15	34.8	7.15	16	35.6	5.04	29	0.435	0.667
STAI-State2 (20–80)	12	34.3	7.39	16	32.9	4.76	25	0.574	0.571
STAI-Trait (20–80)	15	39.9	9.04	16	38.8	5.87	29	0.430	0.671
FAS	15	30.9	22.0	16	22.1	19.9	29	1.18	0.248
		*N*	*%*		*N*	*%*			*p*^a^
Gender [*female*]		13	86.7		12	75.0			0.654

*Means (M), Standard Deviations (SD), df-, t-, and p-Values and also quantity (N) and percentage are given. Note. *df*, degrees of freedom; n, number of participants; Standard Group, extinction after the standard extinction process; Gradual Group, extinction after the gradual extinction process; *STAI-State1, STAI-State2, STAI-Trait*, State (day 1 and 2) and Trait scale of the German version of the State-Trait Anxiety Inventory (Laux et al., 1981), *FAS*, of the German version of the Fear of spiders questionnaire (Szymanski and O’Donohue, 1995) ^a^Fisher’s exact test, two-tailed*.

### Materials

A VR was presented to participants over a V Z800 3D head-mounted display (HMD; eMagin, NY, USA) and was generated with the help of Steam Source engine (Valve Corporation, Bellevue, WA, USA). “Cybersession” software (VTplus GmbH, Würzburg, Germany) controlled the presented VR environment. The participant’s head position was monitored with a Patriot electromagnetic tracking device (Polhemus Corporation, Colchester, VT, USA), which adjusted the field of view in response to head movements. Sounds and instructions were presented over headphones (Sennheiser HD-215, Sennheiser electronic GmbH, Germany). Physiological data were monitored, digitally amplified (V-Amp 16, Brain Products GmbH, Gilching, Germany) and recorded (Brain Vision Recorder software, Version 1.20, Brain Products GmbH, Germany).

**Figure 1:**
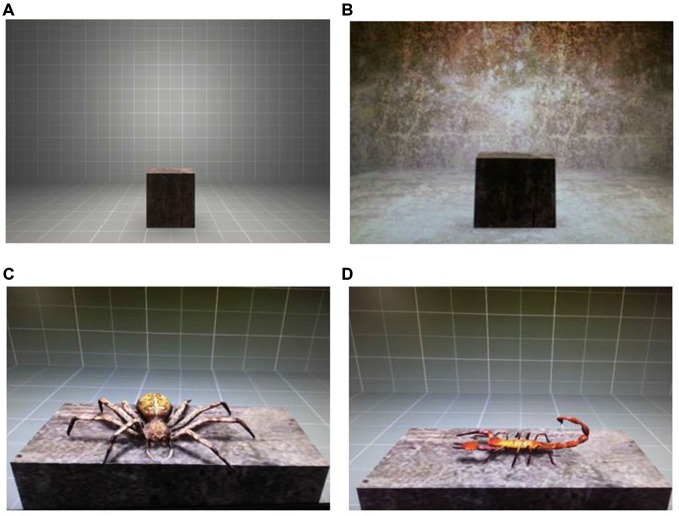
**Virtual environment and virtual stimuli. (A)** Virtual room where acquisition and extinction phases in a virtual reality (VR) took place. **(B)** Virtual room where reinstatement test in VR took place. **(C)** The presented virtual spider was used as an aversive conditioned stimulus (CS+). **(D)** The virtual scorpion was used as a non-aversive CS−.

The VR environment consisted of two rooms, which differed in the textures used for floor, walls and ceiling color (see Figures 1A,B). Participants were able to explore these rooms by looking around, but were unable to move freely. Three stimuli were used for the experiment: one US and two CS. The US involved an air blast (5 bar, 50 ms) aimed at the participant’s right anterior neck. A compressed tank of air was regulated via a magnetic valve system channeling the air through a tube, which was adjusted to the participant’s torso. The CS-US contingency was set at 80% for the acquisition phase. The CS were two virtual animals (virtual spider and scorpion, see Figures 1C,D). They were both presented sitting on a gray platform in the middle of the virtual room during the different phases of the experiment: acquisition, extinction, and reinstatement test. For the CS+, a virtual spider—sitting on the platform and moving its legs—was presented to the participants. The CS− was a scorpion sitting sideways on the platform and moving its tail.

### Measures

Before the VR experiment, participants filled in a demographic questionnaire (age, gender, occupation, and exclusion criteria), and the State-Trait Anxiety Inventory (STAI; Spielberger et al., 1970; German: Laux et al., 1981), which is a commonly used measure for assessing temporary anxiety (state) as well as general anxiousness (trait). Both forms of anxiety are represented by 20 items (statements) each. Answers are given on a four-point Likert scale (state: from 1 = *not at all* to 4 = *very much*; trait: from 1 = *almost never* to 4 = *almost always*). As the State version targets current anxiety caused by the situation at hand, it is filled in on day 1 and 2, while the Trait version for general anxiousness is completed on day 1. For the German version of the STAI (Laux et al., 1981), objectivity concerning conductance, scoring, and interpretation is given. Internal consistency (Cronbach’s Alpha) lies between 0.90 and 0.94 (state) and 0.88 and 0.94 (trait). The retest-reliability coefficient for trait anxiety is between 0.68 and 0.96. Convergent and divergent validity were tested with several populations and were established (Laux et al., 1981). To assess spider phobia, the German version of the Fear of Spiders questionnaire (FSQ; Szymanski and O’Donohue, 1995; German version: FAS; Rinck et al., 2002) with 18 items (which are evaluated on a seven-point Likert scale ranged from 0 = “*I do not agree at* *all*” to 6 = “*I completely agree*”) was used. The translated FSQ demonstrates very high internal consistency, Cronbach‘s Alpha = 0.97, and retest reliability, r_tt_ = 0.95 (Rinck et al., 2002). It is a sensitive measure used to differ between phobics and non-phobics (Szymanski and O’Donohue, 1995).

To measure the emotional state of the participants upon presentation of the virtual animals during different phases, participants were requested to verbally rate the probability that a negative event would occur (contingency rating). The rating scale ranged from 0 (no probability of a negative event occurring) to 10 (100% probability of a negative event occurring), and the ratings were reported at the beginning and at the end of each phase.

Apart from the subjective ratings, two different physiological values were measured. For the startle response, the muscle activity of the Orbicularis Oculi, called the Startle Reflex, was induced by a random noise (white noise: 50 ms, 103 dB), which was presented binaurally over headphones during the presentation of the conditioned stimuli with a contingency of 80%. The reflex was measured with four electrodes (Ag/AgCl, *Ø* = 8 mm) affixed with electrode paste (Signa Creme, Parker Laboratories, New Jersey, USA: Parker Laboratories). Two electrodes were placed under the right eye of the participant and one behind each ear at the mastoid bone for reference and grounding. Impedance level was kept below 5 kΩ.

For the SCR, two electrodes (Ag/AgCl, *Ø* = 8 mm) were attached with electrode cream to the thenar muscle of the non-dominant hand (TD – 246, PAR Medizintechnik GmbH). The skin was cleaned with alcohol prior to electrode attachment.

### Procedure

**Figure 2:**
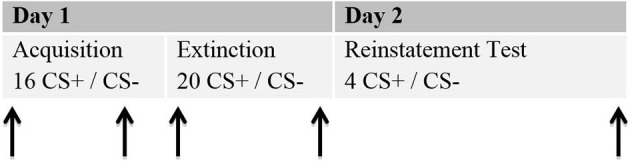
**Schematic procedure of the experiment**. Each phase for the 2 days is given. Arrows represent the moments ratings were given. Each rating included a presentation of the CS+ and the CS−. Stimulus presentations for the ratings are not included in the numbers of CS+ and CS−.

The study was conducted in two sessions, which ran on two consecutive days. An interval of at least 24 h was planned between the sessions, since that is the standard in human fear recovery experiments (Shiban, 2013). Session 1 (about 120 min) involved the acquisition and extinction phase. During Session 2 (about 30 min) the return of fear was tested by a reinstatement test (see Figure 2).

**Figure 3:**
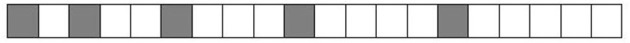
**Pattern of the presentation of the US during the extinction phase for the gradual group**. Each box symbolizes the presentation of a CS+, which was paired with an US at the colored boxes.

At the first day, after the participants filled in the declaration of consent and the questionnaires (demographic, STAI and FAS), the electrodes were adjusted for the physiological measurements, along with the tube for the air puff, the headphones and the HMD. At the beginning of the experiment, there was a short introduction of the procedure and participants were asked to relax for 2 min to assess a baseline for the physiological data. Subsequently, the startle noise was presented repeatedly for a time span of 109 s to prevent distortion of the data caused by habituation to the startle noise. “Habituation is the decline of the acoustic startle response magnitude following repeated presentation of startling stimuli within a single test session” (Koch, 1999). Afterwards, an acclimation phase was initiated, in which all the stimuli we use in this experiment were shortly presented and the participants were instructed to look around the room using head orientation. This was conducted in order to avoid biases in the data caused by context or stimulus novelty effects. The experiment began directly after this phase. The experiment on the first day consisted of the acquisition and extinction phases, both beginning and ending with a rating of contingency. The stimuli were presented in trials, each trial took 30 s and consisted of an eight-second stimulus presentation followed by a 22 s inter-stimulus interval, during which the participant saw a black screen. During the acquisition phase, each stimulus was presented 18 times. The virtual scorpion (CS−) and the virtual spider (CS+) were presented to the participants for 8 s each, and 6 s after the appearance of the virtual animals, a startle noise was presented with a probability of 75% for both stimuli. The CS+ was followed by an aversive air puff (5 bar, 50 ms) 2 s after the appearance of the virtual animals in 80% of all cases, except during the rating phases. After a 10 min break, the experiment continued with the extinction phase, during which the CS+ and CS− were presented 22 times each. The two experimental groups differed as follows. For the SE group, there was no presentation of the US during the extinction phase. For the GE group, the presentation of the aversive air puff following the CS+ was gradually decreased during the extinction phase (see Figure 3). The startle noise continued to emerge as it had before in the acquisition phase, and there was no air puff following the CS−.

At the second day, after the participants completed the STAI State questionnaire for session two, the electrodes, as well as the belt for the air blast tube, were attached at the designated places, and headphones and HMD were adjusted. The session on day 2 consisted of an acclimation phase, similar to day 1, and the reinstatement test. The contingency rating was given in the end of reinstatement test, which took place in a new room (room 2). Reinstatement test started with two presentations of the air puff without showing a stimulus, followed by five CS+ and CS− appearances without the US. The startle noise appeared with a contingency of 75% during the presentation of the CS. The experiment was completed with a final extinction phase that consisted of eight presentations of the spider in room one, without any aversive stimulus or startle noise.

### Experimental Design

**Figure 4:**
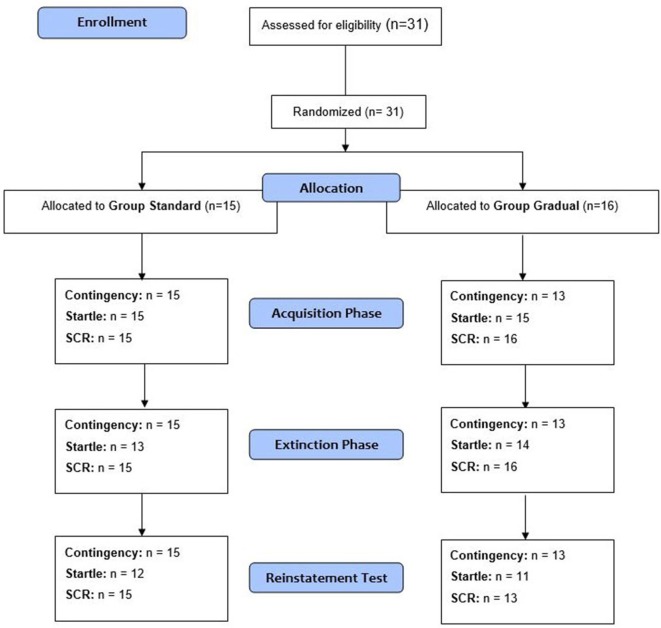
**Schematic procedure**. The number of analyzed data for the measures in each phase of the experiment (acquisition phase, extinction phase, and reinstatement test) is given. Note: n, number of participants with analyzable data.

In accordance with the fear conditioning study by Acheson et al. (2013), the experiment consisted of three phases: acquisition, extinction learning, and extinction recall, which was measured by a reinstatement test. The CS+, one of the two conditioned stimuli, was paired with the US. There was no presentation of the US together with the CS−. The two experimental groups were formed through random assignment of the participants (see Figure 4). Both groups completed all three phases of the experiment. The SE and GE groups differed in their processes during the extinction learning phase. Subjective ratings of contingency as well as the physiological reactions (Startle, SCR) represent the dependent variables.

### Data Reduction and Statistical Analysis

Analyses focused on the participant’s physiological arousal and subjective reactions to the presentation of the CS+ and CS− in the different phases of the experiment in VR: acquisition, extinction and return of fear as tested by reinstatement.

In all three phases, the between-group factor was measured for the Extinction group. The within-group factors stimulus (CS+ vs. CS−) and time were also measured for the different phases: acquisition and extinction (beginning vs. end), and reinstatement (end of extinction vs. reinstatement).

Physiological data were preprocessed with Brain Vision Analyzer 2.0 software (Brain Products GmbH, Munich, Germany) and further analyses were performed in SPSS 22.0 (IBM Corp., Armonk, NY, USA).

For the startle response, at first, differences between the two EMG electrodes were computed (see Blumenthal et al., 2005). A 249 Hz high cut-off filter, a 28 Hz low cut-off filter and a 50 Hz notch filter were applied. The data were rectified, and a moving average (50 ms) was calculated. For each startle, a baseline correction was conducted using the mean value of the 50 ms before each startle tone as baseline. Next, peaks were marked automatically and manually controlled and corrected if necessary. Finally, *T*-values for the startle magnitude were calculated.

For SCR, a 1 Hz cut-off filter was applied. Data were rectified and for each SCR, a baseline correction was conducted using the mean value of the 500 ms before each presentation of the stimulus as a baseline. For peak detection, data from 3000 to 6000 ms after the presentation of the stimuli were segmented. Peaks were marked automatically and manually controlled and corrected if necessary. Finally, *T*-values for the SCR were calculated.

For physiological outcome variables in the acquisition and extinction phases, physiological data of the first four (beginning) and last four (end) presentations of the stimuli in each phase were used to calculate means. For the reinstatement test, means were calculated with the data following four stimuli presentations. For each outcome variable (contingency ratings, startle response, SCR) that was measured in the two rooms, means for CS+ and CS− were calculated.

For contingency ratings, startle and SCR repeated-measures ANOVAs with the within-subjects factor time (Beginning vs. End), stimulus (CS+ vs. CS−) and between-subjects factor group (SE vs. GE) were applied for each phase (acquisition and extinction and reinstatement test).

In additional analyses of significant effects of time, stimulus or group Student’s *t*-tests were performed. Partial η^2^ (${\text{η}}_{p}^{2}$) scores and Cohen’s *d* were used as indices of effect size. The significance level was set at two-tailed α = 0.05.

## Results

### Acquisition

#### Contingency Ratings

**Figure 5:**
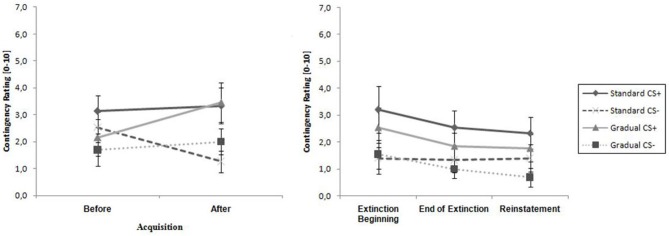
**Contingency rating for CS+ and CS− for all phases for the Gradual and Standard groups**. Note: CS+, stimulus with negative consequences; CS−, stimulus without negative consequences; Standard, the experimental group which participated in the Standard extinction (SE) process; Gradual, the experimental group which participated in the Gradual extinction (GE) process. Mean contingency ratings are given. Standard errors are presented as error bars.

As visible in Figure 5, the contingency ratings for the CS+ were higher than the ratings for the CS− before the acquisition phase, as well as afterwards, and increased in both groups over time, while the CS− ratings either increased minimally (GE) or decreased (SE). An ANOVA revealed a significant main effect of stimulus, *F*_(1,26)_ = 13.8, *p* < 0.001, ${\text{η}}_{p}^{2}$ = 0.35, as well as an interaction effect of Time × Stimulus, *F*_(1,26)_ = 11.3, *p* < 0.002, ${\text{η}}_{p}^{2}$ = 0.30. Follow-up on the significant interaction effect demonstrated that at the end of the acquisition phase the CS+ and CS- differed significantly, *t*_(26)_ = 4.51, *p* < 0.001, *d* = 0.84. Means and standard deviations can be viewed at Table 2. These results indicate that successful acquisition took place.

**Table 2:** **Contingency ratings, startle response and skin conductance response for CS+ and CS−**.

		Standard group					Gradual group			
		CS+		CS−			CS+		CS−	
Phase		*M*	*SD*	*M*	*SD*	*p*	*M*	*SD*	*M*	*SD*	*p*
*Contingency ratings*
Acquisition	Start	3.13	2.26	2.53	2.20	0.237	2.15	2.44	1.69	2.18	0.235
	End	3.33	2.58	1.27	1.53	0.002	3.46	2.65	2.00	1.73	0.028
Extinction	Start	3.20	3.36	1.40	2.23	0.022	2.54	2.60	1.54	1.94	0.121
	End	2.53	2.50	1.33	1.80	0.012	1.85	1.82	1.33	1.23	0.136
Reinstatement		2.33	2.29	1.40	1.99	0.001	1.77	1.83	0.69	1.25	0.025
*Startle response*
Acquisition	Start	58.5	8.30	53.0	5.55	0.039	59.9	5.73	53.4	7.22	0.010
	End	49.7	7.60	48.1	3.57	0.446	52.9	5.60	47.2	3.61	0.009
Extinction	Start	50.6	4.56	48.8	5.48	0.107	52.6	5.53	49.4	6.37	0.203
	End	47.1	4.08	44.6	2.70	0.052	46.2	2.56	45.4	4.76	0.413
Reinstatement		51.6	8.34	46.7	6.34	0.004	46.3	6.11	46.6	10.2	0.644
*Skin conductance response*
Acquisition	Start	49.7	5.54	48.1	4.66	0.476	52.4	6.84	48.1	4.06	0.024
	End	52.3	4.73	49.9	5.62	0.305	51.1	5.14	50.0	5.27	0.347
Extinction	Start	48.0	2.44	49.3	3.54	0.285	48.1	5.14	49.4	1.91	0.420
	End	50.0	6.70	51.5	2.83	0.499	49.6	3.32	52.0	5.00	0.079
Reinstatement		49.0	4.36	46.8	4.24	0.121	49.5	5.13	47.3	3.82	0.158

*Means (M), Standard Deviation (SD) and p-Values of the contingency ratings, startle response and skin conductance response are given. Note: Standard Group, the experimental group which participated in the Standard Extinction (SE) process; Gradual Group, the experimental group which participated in the Gradual Extinction (GE) process; n, number of participants; CS+, stimulus (spider) with aversive unconditioned stimulus (US); CS−, stimulus (scorpion) without aversive US. For the contingency ratings in all three phases were n = 15 in the SE and n = 13 in the GE. For the startle response, in the acquisition were n = 15 in both groups, in the extinction were n = 13 in the SE and n = 14 in the GE, and in the reinstatement test were n = 12 in SE and n = 11 in GE. For the skin conductance response, in the acquisition and extinction were n = 15 in SE and n = 16 in GE, and in the reinstatement test were n = 15 in SE and n = 13 in GE*.

#### Startle Response

The two experimental groups did not differ at the beginning or end of this phase. The CS+ caused a higher reaction than the CS− during the entire phase, and the response decreased from the beginning to the end of acquisition phase for both groups (see Table 2). This pattern was also reflected only in a significant main effect of time, *F*_(1,28)_ = 23.4, *p* < 0.001, ${\text{η}}_{p}^{2}$ = 0.46 and stimulus, *F*_(1,28)_ = 21.7, *p* < 0.001, ${\text{η}}_{p}^{2}$ = 0.43 in the acquisition phase.

#### Skin Conductance Response

There were similar SCR levels in the two experimental groups at the beginning and the end of the acquisition phase, as shown in Figure 7. For the whole samples, the CS+ triggered a higher SCR than the CS− during the acquisition phase (see Table 2), underlined by a significant main effect of stimulus, *F*_(1,29)_ = 6.60, *p* = 0.016, ${\text{η}}_{p}^{2}$ = 0.19. These results indicate that the electrodermal activity did not change significantly with time.

**Figure 6:**
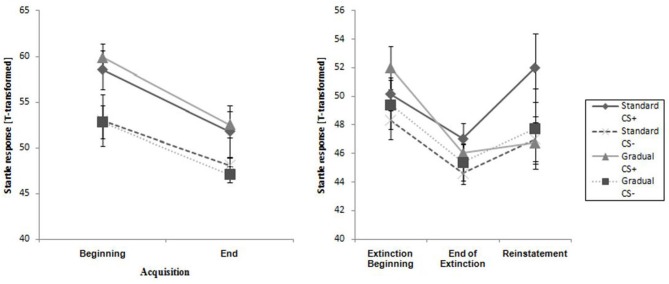
**Startle response for CS+ and CS− for all phases for the Gradual and Standard groups**. *Note:* CS+, stimulus with negative consequences; CS−, stimulus without negative consequences; Standard, the experimental group which participated in the Standard Extinction process; Gradual, the experimental group which participated in the Gradual Extinction process. Mean startle responses are given. Standard errors are presented as error bars.

**Figure 7:**
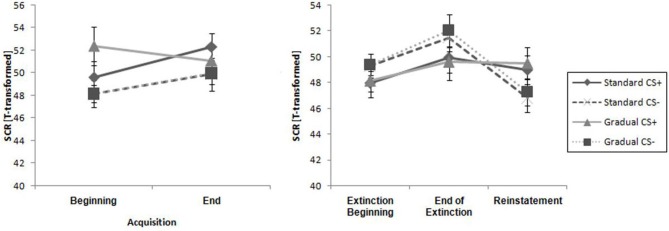
**Comparison of SCR at all phases for the gradual and standard groups**. Note: CS+, stimulus with negative consequences; CS-, stimulus without negative consequences; Standard, the experimental group which participated in the Standard Extinction process; Gradual, the experimental group which participated in the Gradual Extinction process. Mean startle responses are given. Standard errors are presented as error bars.

### Extinction

#### Contingency Ratings

As shown in Table 2, the CS+ was rated higher than the CS− by both groups at the beginning as well as at the end of the extinction phase, which was confirmed by a significant main effect of stimulus, *F*_(1,26)_ = 10.6, *p* < 0.003, ${\text{η}}_{p}^{2}$ = 0.29. There were no further effects.

#### Startle Response

As Figure 6 demonstrates, the startle response decreased from the beginning to the end of the extinction phase in both groups (see Table 2), which is emphasized by a significant main effect of time, *F*_(1,25)_ = 27.3, *p* < 0.001, ${\text{η}}_{p}^{2}$ = 0.52. The main effect of stimulus, *F*_(1,25)_ = 5.19, *p* = 0.031, ${\text{η}}_{p}^{2}$ = 0.17 shows it is evident that there was a higher startle response caused by the CS+ for both groups during the extinction phase.

#### Skin Conductance Response

As it can be seen in Table 2, the response increased over time and the CS− caused a higher level of activity than the CS+ during the course of the whole period, which was reflected by a main effect of time, *F*_(1,29)_ = 8.27, *p* = 0.007, ${\text{η}}_{p}^{2}$ = 0.22, and stimulus, *F*_(1,29)_ = 5.02, *p* = 0.033, ${\text{η}}_{p}^{2}$ = 0.15. Means and standard deviations can be seen in Table 2. These results do not suggest there was successful extinction.

### Return of Fear: Reinstatement

#### Contingency Ratings

For both groups, the ratings of the CS+ are significantly higher than for the CS−, which is shown by a main effect of stimulus, *F*_(1,26)_ = 12.0, *p* = 0.002, ${\text{η}}_{p}^{2}$ = 0.32. No other effects were significant. The ratings remained constant during the period of time when the end of the extinction phase and the reinstatement test are compared. Means and Standard deviations can be seen in Table 2. No return of fear was noticeable from the contingency ratings during the reinstatement test.

#### Startle Response

As shown in Figure 6, the startle response during the reinstatement test tended to be higher than at the end of the extinction phase for each group and stimulus. The CS+ caused a higher startle response than the CS− for both experimental groups at both times. An ANOVA confirmed a significant main effect of stimulus, *F*_(1,21)_ = 6.98, *p* = 0.015, ${\text{η}}_{p}^{2}$ = 0.25) and a significant Stimulus x Group interaction, *F*_(1,21)_ = 5.39, *p* = 0.030, ${\text{η}}_{p}^{2}$ = 0.21. In order to follow up on the group-related interactions, a separate ANOVA for each of the two groups was conducted. For group SE, a significant main effect of stimulus, *F*_(1,11)_ = 30.5, *p* < 0.001, ${\text{η}}_{p}^{2}$ = 0.735 was found. Follow-up *t*-tests for the startle response showed that the CS+ was significantly higher than the CS−, *t*_(11)_ = 30.5 *p* < 0.001, *d* = 0.69) for the SE group. For the GE group, an ANOVA showed no significant effects. These results indicate that, according to the startle response, more return of fear took place for the SE than the GE group. Table 2 shows means and standard deviations.

#### Skin Conductance Response

The SCR at the end of the extinction phase was higher than at the reinstatement test, underlined by the main effect of time, *F*_(1,26)_ = 9.93, *p* < 0.004, ${\text{η}}_{p}^{2}$ = 0.28 (see Table 2). Furthermore, there were no significant differences between the groups or the stimuli. There is no return of fear that can be proven by the SCR data.

## Discussion

The main goal of this study was to apply the findings from Gershman et al. (2013) to a human sample in order to support the notion that GE is a successful method for preventing the return of fear following an extinction procedure. We were able to achieve similar results for the startle variable in the reinstatement test but not for the SCR and contingency rating in our human sample.

In our study, participants showed *acquisition effects* as reflected in a higher response towards the CS+ compared to the CS− in the contingency and some of the physiological measures. In the startle and SCR response there was a significant difference between the two stimuli over the whole acquisition period. This was expected because the measures at the beginning of the acquisition were conducted in the first four presentations of the acquisition phase and not before the acquisition. The analysis of the contingency ratings reflected a significant difference between the two stimuli only after the acquisition but not before. This was expected as well because the contingency ratings were measured pre- and post-acquisition. Furthermore, as expected, there were no group differences in this phase.

As for the *extinction phase*, we could see an inhibition effect during extinction in the form of a significant reduction of the startle response. However, this was not restricted to the CS+, the CS− also showed a clear inhibitory effect. Surprisingly, the SCR showed an increase in arousal through the extinction phase (in all stimuli in both groups). The contingency data shows that the CS+ contingency scores were reduced (unlike the CS− scores) but this reduction did not reach significance, possibly due to the small sample size we used. An alternative explanation might be the fact that the US did not induce a strong fear reaction. This was an unexpected result which contrasts with the results of the startle response. Interestingly, there was no significant difference in the extinction phase between the groups even though one of the groups was partly exposed to the US. This is consistent with the results from the study by Gershman et al. (2013).

During the *reinstatement* phase in the startle response, there was similarly a return of fear reaction in the reinstatement test; however, this effect was evident only for the SE group, just as we expected. The SCR values decreased significantly when comparing the end of the extinction phase and the reinstatement test and there was no significant difference between the two stimuli during the whole phase. Contingency ratings were higher for the CS+ compared to the CS−, but no group differences were evident.

Overall, it was evident from the startle response data that there were less fear responses in the GE group than in the SE group. This corresponds to the results from the study by Gershman et al. (2013), who also found a reduction of the fear response after GE. It is worth mentioning that the dependent variable used in their study was freezing reaction.

Based on this result we suggest that the reduction of the return of fear caused by GE is not restricted to animals but can also be seen at least partly in humans, too. This is an important issue when considering the transfer of results from laboratory research to clinical practice. Extinction serves as the laboratory counterpart of exposure therapy (Hermans et al., 2006). Replication of this effect in further studies could have major implications for the practical treatment of fear related disorder. For example, in social phobia one method is exposure in which a patient is asked to hold a public speech in front of an audience (Anderson et al., 2005). An incorporation of gradual exposure may suggest to gradually reduce the aversive reaction from an audience during exposure and not to expose a participant to a continually friendly audience. A further strength of our paradigm is the use of VR to achieve a high level of standardization during our paradigm. We also wish to emphasize the fact that we measured fear on multiple levels by using startle, SCR and contingency ratings. However, some limitations must be taken into account. Firstly, because not all measurements showed a clear acquisition and extinction effect, we suggest the use of a stronger conditioning procedure (stronger in the sense of being more aversive) as we used an air puff of only 5 bar as US. One possibility is replacing it with electric stimulation. Another advantage of using an electrical stimulation as US is the link between the US and the spider (CS+) because the US can mimic a spider bite. It is also important to discuss the unexpected results from the contingency ratings, for we didn’t find significant shifts of the CS+ or the CS− during the extinction or reinstatement phases. We asked the participants to rate the probability that a negative event would occur. An improvement would be to specify this question and to ask them directly to rate the probability that an air puff will occur. Another option would be to increase this contingency from 80–100%. This, however, might influence the extinction procedure, as partial reinforcement leads to a learning effect that is robust to extinction and though slowing the extinction effect (Atkinson et al., 1995). So increasing the expectancy of the US during acquisition might cause an increased and fast extinction effect.

We found conflicting results from the startle and the SCR measures. As Hamm and Weike (2005) pointed out, startle is sensible for measuring fear learning independently from contingency awareness, but SCR requires contingency awareness learning. As we found no extinction in the contingency data we believe this might explain at least partly the lack of effect on the SCR data. We believe that improving the paradigm so that the contingency ratings will reflect a clear extinction and reinstatement effects will probably lead to similar effects in the SCR data. Importantly, our study aimed at transferring the findings from Gershman et al. (2013), who employed “freezing” measures to evaluate fear reaction in mice, to a human sample. As Leaton and Borszcz (1985) discuss, startle (in humans) and freezing (in mice) are two highly related measures. This is especially important as startle in humans is also related to learning without cortical involvement. Therefore, it is plausible that we found significant results for the startle measures, too, but not for the SCR measures.

The sample size of this study must also be acknowledged as further limitation. It consisted of 31 participants altogether. More distinctive results should be expected with a larger sample size.

Furthermore, it could be argued that a longer interval between the acquisition and extinction phase might ensure that participants perceive the two phases as separate stages. Using short intervals as we did might inhibit the consolidation of the long-term memory (Myers and Davis, 2007). Future studies might investigate if there is a clearer effect in the extinction and return for fear when the extinction phase follows an extended time interval.

As represented in the introduction, there are many different theories describing the reasons why fear returns after extinction. One prominent theory states that presenting some USs during the extinction phase prevents the formation of a new “state”. Participants do not learn to create a new CS-no-US-state (Bouton, 2004) but realize instead that there is no fearful event following the CS+. As a result, they no longer perceive the CS+ as fearful, thereby transforming the original fear memory into a no fear memory. A different approach suggests that presenting the US (prior to extinction) serves as a signal which reactivates the fear structure. This renders the memory into a labile state which enables modifications (Schiller et al., 2010). It would be interesting to investigate whether GE can induce reactivation of the fear memory similar to what Schiller et al. (2010) suggest, thereby causing fear memory to be extinguished.

In summary, GE seems to be a better alternative to the SE process because it prevents the return of fear. Future studies are needed to replicate and extend this effect. Especially interesting would be the question whether this effect can be seen in other measures than the startle response. A long term goal would be to test the effect of GE in a clinical sample. This could lead to important improvements to the structure of exposure therapy in treating patients with anxiety disorders.

## Author Contributions

YS, study conception, data analysis, wrote the manuscript. JW, study conception, data acquisition and analysis, and contribution to the manuscript. MW, study conception, data acquisition and analysis, and contribution to the manuscript. AM, study conception, data analysis, and contribution to the manuscript. All authors have approved of the final version of the manuscript and its submission.

## Conflict of Interest Statement

Andreas Mühlberger is stakeholder of a commercial company that develops virtual environment research systems. The other authors declare that the research was conducted in the absence of any commercial or financial relationships that could be construed as a potential conflict of interest.
